# Insulin Resistance and Chronic Kidney Disease in Patients with Type 1 Diabetes Mellitus

**DOI:** 10.1155/2017/6425359

**Published:** 2017-03-14

**Authors:** Mihaela Vladu, Diana Clenciu, Ion Cristian Efrem, Mircea-Cătalin Forțofoiu, Anca Amzolini, Simona Tudorică Micu, Maria Moţa, Maria Forțofoiu

**Affiliations:** ^1^University of Medicine and Pharmacy of Craiova, Craiova, Romania; ^2^Clinical Municipal Hospital “Philanthropy” of Craiova, Craiova, Romania

## Abstract

*Background and Aims*. Diabetes mellitus (DM) is a chronic disease which can evolve towards devastating micro- and macrovascular complications. DM is the most frequent cause of chronic kidney disease (CKD). Insulin resistance plays an important role in the natural history of type 1 diabetes. The purpose of the study was to determine the prevalence of CKD in T1DM and the correlation with insulin resistance (IR) in patients with CKD.* Materials and Methods*. The study was conducted over a period of three years (2010–2013) and included patients with DM registered in the Clinical Centre of Diabetes, Nutrition and Metabolic Diseases of Dolj county. The study design was an epidemiological, transversal, noninterventional type. Finally, the study group included 200 subjects with type 1 DM. Insulin resistance (IR) was estimated by eGDR. The subjects with eGDR ≤ 7.5 mg/kg/min were considered with insulin resistance.* Results*. CKD was found in 44% of the patients. Analyzing statistically the presence of CKD, we found highly significant differences between patients with CKD and those without CKD regarding age and sex of the patients, the duration of diabetes, glycosylated hemoglobin (HbA1c), the estimated glucose disposal rate (eGDR), and the presence of hypertension, dyslipidemia, and hyperuricaemia. In patients with CKD, age and diabetes duration are significantly higher than in those who do not have this complication. CKD is more frequent in males than in females (50.9% men versus 34.5% women, *p* = 0.022). From the elements of metabolic syndrome, high blood pressure, hyperuricemia, and dyslipidemia are significantly increased in diabetic patients with CKD. eGDR value (expressed as mg·kg^−1^·min^−1^) is lower in patients with CKD than in those without CKD (15.92 versus 6.42, *p* < 0.001) indicating the fact that patients with CKD show higher insulin resistance than those without CKD.* Conclusions.* This study has shown that insulin resistance is associated with an increased risk of CKD, but, due to the cross-sectional design, the causal relationship cannot be assessed. However, the existence of this causality and the treatment benefit of insulin resistance in type 1 diabetes are issues for further discussion.

## 1. Background and Aims

Diabetes mellitus (DM) is a chronic disease which can evolve towards devastating micro- and macrovascular complications. DM is the most frequent cause of chronic kidney disease (CKD). The diabetic chronic kidney disease (CKD) is a clinical syndrome characterized by persistent albuminuria (albumin/creatinine ratio in the spontaneous urine ≥ 30 mg/g) and/or a sustained decline of the estimated glomerular filtration rate (eGFR) below 60 mL/min/1.72 m^2^. If at least one of these values is still maintained within these abnormal limits after 3 months from the first measurement, the diagnosis of diabetic CKD may be established [1, 2].

Insulin resistance has been linked to the pathophysiology of type 2 diabetes mellitus (T2DM) and it has been proven to play an important role in the increase of the risk of cardiovascular complications. It has been recently shown that insulin resistance plays an important role in the natural history of type 1 diabetes, although it is generally known that it is mainly due to immune destruction of the *β*-pancreatic cells [3].

Since 1977, Ginsberg has studied the association of insulin resistance with type 1 diabetes mellitus (T1DM) [4]. Subsequent studies [5, 6] showed a significant decrease in glucose disposal mediated by insulin among patients with T1DM suggesting the presence of insulin resistance. After a decade, this combination has aroused the interest of many research studies [7, 8] to investigate the possible mechanisms of insulin resistance in T1DM. Insulin resistance is a feature present in T1DM [9–11]; clamp studies on T1DM confirm its growth in patients with microalbuminuria [12] while glomerular filtration rate decreases [13]. Insulin resistance is a progressive process increasing the urinary excretion of albumin and decreasing glomerular filtration rate [14].

The purpose of the study was to determine the prevalence of CKD in T1DM and the correlation with insulin resistance (IR) in patients with CKD.

## 2. Materials and Methods

The study was conducted over a period of three years (2010–2013) and was comprised of patients with DM registered in the Clinical Centre of Diabetes, Nutrition and Metabolic Diseases of Dolj county.

The study design was an epidemiological, transversal, noninterventional type. Finally, the study group included 200 subjects with T1DM.

Anamnestic data have been analyzed (age, sex, duration of DM, history of arterial hypertension, dislipidemia, and hyperuricemia), as well as paraclinical data (urea, creatinine, uric acid, cholesterol, triglycerides, A1c, and urinary albumin-to-creatinine ratio). The estimated glomerular filtration rate (eGFR) has also been calculated according to the Modification of Diet in Renal Disease (MDRD) equation. The CKD stages have been established according to KDIGO 2012 definition. In predicting the risk for outcome of CKD, we used GFR and albuminuria category. GFR from 60 to 89 mL/min/1.73 m^2^ is classified as CKD stage 2, from 45 to 59 mL/min/1.73 m^2^ as CKD stage 3a, from 30 to 44 mL/min/1.73 m^2^ as CKD stage 3b, and from 15 to 29 mL/min/1.73 m^2^ as CKD stage 4. Stage 1 is defined by GFR over 90 mL/min/1.73 m^2^ and stage 5 by GFR under 15 mL/min/1.73 m^2^. Albuminuria categories were A1 < 30 mg/g (normally to moderately increased), A2 = 30–300 mg/g (moderately increased), and A3 > 300 mg/g (severely increased).

Insulin resistance was evaluated by the estimated glucose disposal rate (eGDR). Subjects with eGDR ≤ 7.5 mg/kg/min were considered with IR. The euglycemic-hyperinsulinemic clamp is the accepted standard for measurement of insulin sensitivity; however, it is not practical for use in the clinical setting. The eGDR can be calculated using routine clinical measures: the glycosylated hemoglobin (HbA1c), the presence of hypertension, and the waist circumference [15, 16]. We used the eGDR because it shows good correlation with IR measured by the euglycemic-hyperinsulinemic clamp and has been validated for the estimation of insulin sensitivity in individuals with type 1 DM.

### 2.1. Statistical Analysis

The recorded data have been analyzed using the Statistical Package for the Social Sciences (SPSS), version 17.00, software (IBM Corporation, Armonk, NY, USA). We performed analysis of the entire study population and separate statistics for each of the 3 groups. The methods used were *t*-test, Mann–Whitney test, Chi-square test, and Cramer test as appropriate. We used the following interpretation of *p* values: *p* < 0.05, the difference between the two means is significant (S); *p* < 0.01, the difference between the two means is highly significant (HS); *p* < 0.001, the difference between the two averages is very highly significant (VHS); *p* > 0.05, the difference between the two means is not significant (NS).

## 3. Results

The study included 200 subjects with type 1 diabetes (116 men and 84 women).  Table 1 summarizes the characteristics of the population studied. Data are presented as mean ± standard deviation.

**Figure 1:**
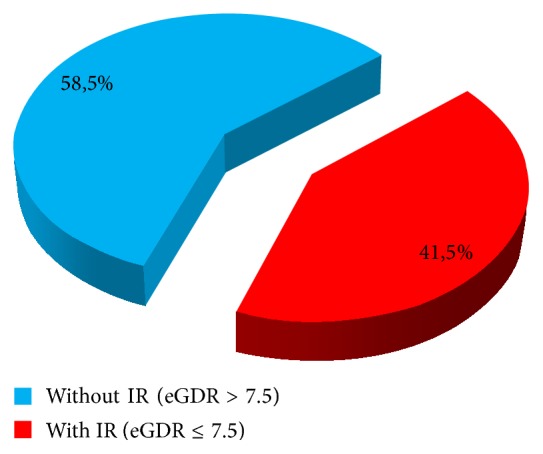
Insulin resistance in type 1 DM patients.

**Figure 2:**
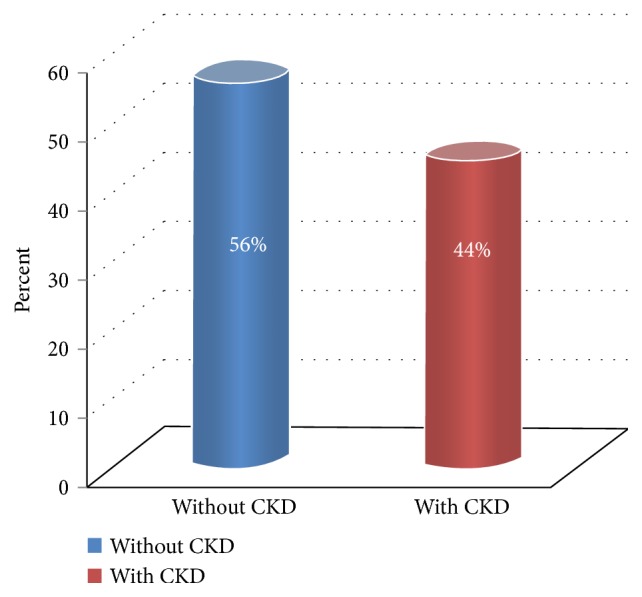
Graphic representation of CKD.

Insulin resistance was found in 41.5% of patients with type 1 DM (Figure 1). CKD was found in 44% of the patients (Figure 2). Statistically analyzing the presence of CKD, there were statistically highly significant differences between the patients with CKD and those without CKD with regard to age and sex of the patients, duration of diabetes, HbA1c, eGDR, and the presence of hypertension, dyslipidemia, and hyperuricaemia (Table 2).

In patients with CKD, the age and diabetes duration are significantly higher than in those who do not have this complication. CKD is more frequent in males than in females (50.9% men versus 34.5% women, *p* = 0.022). High blood pressure, hyperuricemia, and dyslipidemia are significantly increased in the diabetic patients with CKD.

**Figure 3:**
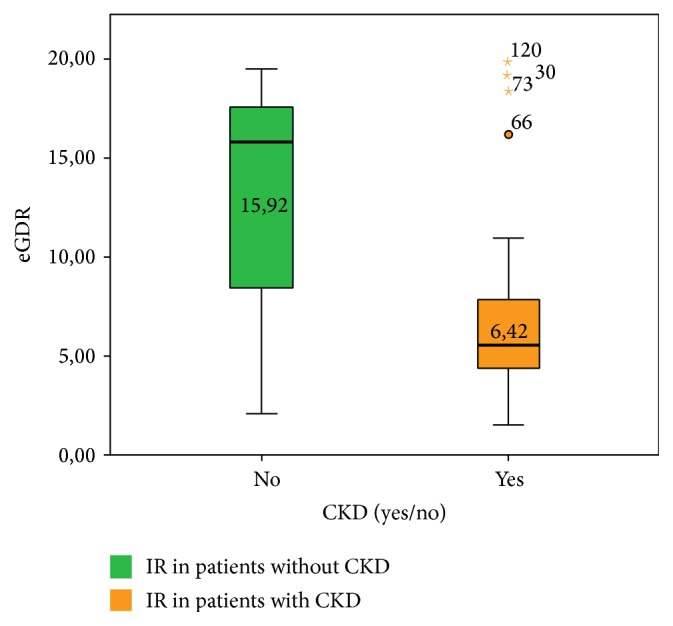
Insulin resistance in CKD patients.

eGDR value (expressed as mg·kg^−1^·min^−1^) is lower in patients with CKD than in those without CKD (15.92 versus 6.42, *p* < 0.001), indicating that patients with CKD show higher insulin resistance than those without CKD (Figure 3).

By performing the logistic regression analysis of the stepwise type where we introduced all statistically significant parameters in Table 2, we obtained the final regression model shown in Table 3.

**Figure 4:**
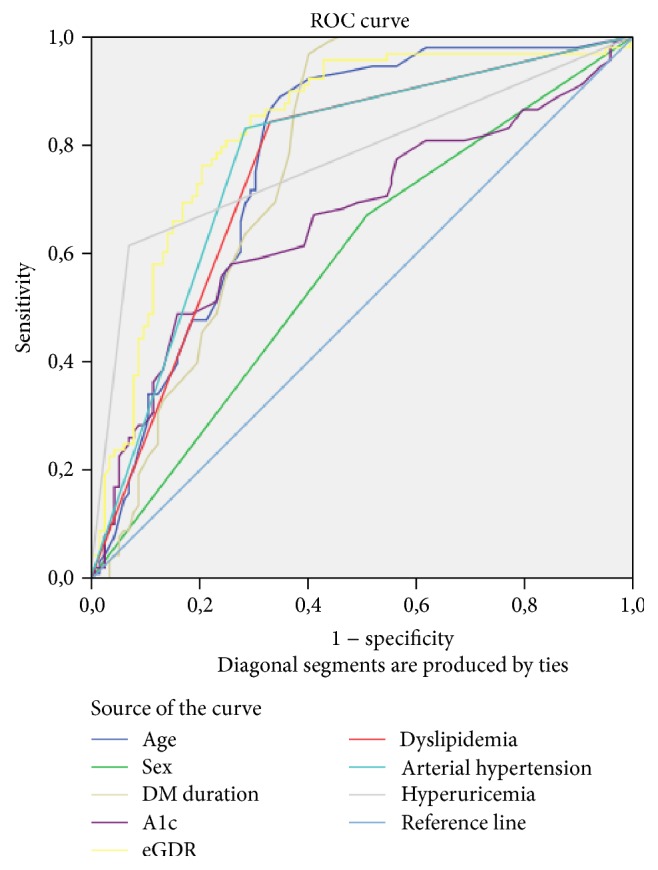
Analysis of the area under the ROC curve for the eGDR in patients with CKD.

Analyzing the area under the ROC curve, used to assess the usefulness of studying the parameters from Table 3 as independent predictors for the occurrence of CKD in patients with type 1 diabetes, the best predictor appears to be the eGDR value followed by the duration of diabetes, age, hypertension, dyslipidemia, and blood pressure (Table 4 and Figure 4).

## 4. Discussions

Older age and long evolution of DM are factors known and shown in numerous studies to be associated with the development and progression of chronic complications in diabetes, whereas this new concept of insulin resistance has appeared lately in type 1 DM and it has gained ground worldwide being accepted by doctors and researchers. Its association with the emergence of chronic vascular complications in particular triggered the development of several studies that try to explain the link between insulin resistance and type 1 DM.

In European countries such as Finland, the country with the highest rate of incidence and prevalence of diabetes type 1 in the world, insulin resistance is associated with older age, longer duration of diabetes, and an increased amount of fat tissue [17] with a family history of type 2 DM, with poor glycemic control [15] and increased serum lipid levels. In Asian countries, a low prevalence of this disease prevented the researchers from investigating the link between insulin resistance and type 1 DM. In general, the prevalence of insulin resistance in the Asian population both in diabetic and in nondiabetic populations is relatively small compared to the Caucasian race, this thing being attributed to lower rates of obesity in Asians.

In this study, we evaluated the prevalence of insulin resistance in patients with type 1 diabetes using clinical score eGDR, its results showing a prevalence of 41.5%, similar to other European countries where the prevalence cited in studies is 32–48%.

As in the studies conducted in Europe, older age, longer duration of diabetes development, hypertension, and dyslipidemia are more frequent characteristics among adults with type 1 DM with insulin resistance.

The small sample of 200 patients used in this study compared with the studies conducted in the European Union may explain some of the differences which appear. In addition, both groups have a large number of subjects under insulin treatment inadequately dosed due to the risk of hypoglycemia especially in the context given by the presence of CKD. The results of this study showed that insulin resistance, measured by eGDR, is more frequent in patients with CKD than in those without CKD.

## 5. Conclusions

- This study has shown that insulin resistance is associated with an increased risk of CKD, but, because of the cross-sectional design, the causal relationship cannot be assessed. However, the existence of this causality and the benefit of treatment for insulin resistance in type 1 DM are issues for further discussion.
- In conclusion, our study has shown that insulin resistance is a constant factor associated with CKD in type 1 DM and it may be useful in the future for CKD screening.

## Figures and Tables

**Table 1:** Characteristics of the studied population.

Parameters	Value
Sex (M/F)	116 (58%)/84 (42%)
Age	37,57 ± 12,45
Age at the onset of DM	21,49 ± 8,49
Duration of DM	16,21 ± 8,97
BMI (kg/m^2^)	23,84 ± 3,72
Abdominal circumference	88,43 ± 10,21
Hip circumference	96,34 ± 7,77
Waist/hip index	0,91 ± 0,07
eGFR	92,39 ± 25,17
eGDR	10,06 ± 5,56
Total cholesterol	170,35 ± 41,61
HDL-cholesterol	61,35 ± 57,87
Triglyceride	114,6 ± 66,68
A1c	8,4 ± 1,58
CKD	44%
Hypertension	52,5%
Dyslipidemia	55,5%
Hyperuricemia	31%

**Table 2:** Characteristics of patients with CKD.

Variables	With CKD	Without CKD	*p*
Number of patients	88 (44%)	112 (56%)	N/A
Age (years)	43,78 ± 10,57	32,68 ± 11,64	*p* < 0,001
Sex (M/F)	59/29	57/55	*p* = 0,022
Duration of DM (years)	20,05 ± 6,62	13,2 ± 9,44	*p* < 0,001
BMI (kg/m^2^)	24,28 ± 3,68	23,49 ± 3,73	*p* = 0,057
AC (cm)	88,88 ± 10,83	88,08 ± 9,73	*p* = 0,671
A1c (%)	8,88 ± 1,71	8,02 ± 1,26	*p* < 0,001
eGDR (mg·kg^−1^·min^−1^)	6,42 ± 3,44	15,92 ± 5,22	*p* < 0,001
Dyslipidemia	59 (67%)	37 (33%)	*p* < 0,001
Hypertension	61 (69,3%)	34 (30,3%)	*p* < 0,001
Hyperuricemia	77 (87,5%)	14 (12,5%)	*p* < 0,001

**Table 3:** The final model of logistic regression for CKD.

	OR	95% CI	*p*
Age	1,086	1,056–1,116	*p* < 0,001
Sex	0,509	0,286–0,909	*p* = 0,022
Duration of DM	1,105	1,062–1,150	*p* < 0,001
A1c	1,450	1,186–1,774	*p* < 0,001
eGDR	0,744	0,684–0,810	*p* < 0,001
Dyslipidemia	10,714	5,354–21,442	*p* < 0,001
Hypertension	12,167	6,099–24,269	*p* < 0,001
Hyperuricemia	20,647	8,937–47,702	*p* < 0,001

**Table 4:** Area under the ROC curve analysis for statistically significant parameters associated with CKD.

	Area under the ROC curve	95% CI
eGDR	**0,829**	0,770–0,888
Age	**0,774**	0,708–0,840
Hypertension	**0,772**	0,705–0,839
Hyperuricemia	**0,771**	0,701–0,841
Duration of DM	**0,770**	0,704–0,836
Dyslipidemia	**0,755**	0,687–0,824
A1c	0,663	0,585–0,741
Sex	0,581	0,501–0,660
